# A Participatory Investigation of Bovine Health and Production Issues in Pakistan

**DOI:** 10.3389/fvets.2020.00248

**Published:** 2020-05-06

**Authors:** Abdul Ghafar, David McGill, Mark A. Stevenson, Muhammad Badar, Aijaz Kumbher, Hassan M. Warriach, Robin B. Gasser, Abdul Jabbar

**Affiliations:** ^1^Department of Veterinary Biosciences, Faculty of Veterinary and Agricultural Sciences, Melbourne Veterinary School, The University of Melbourne, Werribee, VIC, Australia; ^2^Department of Veterinary Clinical Sciences, Faculty of Veterinary and Agricultural Sciences, Melbourne Veterinary School, The University of Melbourne, Werribee, VIC, Australia; ^3^Livestock and Dairy Development Department, Lahore, Pakistan; ^4^Dairy Beef Project, University of Veterinary & Animal Sciences, Lahore, Pakistan

**Keywords:** agroecological zones, bovine health, Pakistan, participatory epidemiology, production, small-scale dairy farming

## Abstract

Systems to record the frequency of animal health events in Pakistan are limited. A participatory approach was used to address gaps in farmers' knowledge and understanding of bovine health and production issues in five agroecological zones (AEZs) of Pakistan. Participatory tools, including simple ranking, pairwise ranking, constraint impact scoring, and constraint profiling were used in group discussions with farmers and animal health professionals (AHPs) in six districts of two provinces, Punjab and Sindh. The results of the ranking activities showed that foot-and-mouth disease (FMD), clinical mastitis, ticks, hemorrhagic septicemia, reproductive disorders, blackleg, and endoparasites were the most important bovine health and production constraints for small-scale dairy farmers. Constraint impact scoring showed that the participants perceived that: (1) milk production was severely affected by FMD and mastitis; (2) blackleg and parasitism led to poor growth rates and reduced meat production; (3) reproductive disorders and mastitis caused major economic losses (due to the high cost of treatment); and (4) blackleg and hemorrhagic septicemia were the leading causes of mortality in cattle and buffaloes. Although there was strong agreement in responses and constraint impact scores between farmers and AHPs, farmers were more concerned about health issues that cause high mortalities, whereas AHPs emphasized the importance of disorders with a high economic impact. Despite socioeconomic differences among AEZs, farmers' knowledge about bovine health and production constraints was similar. The findings from this study revealed that farmers had limited understanding of the risk factors and routes of transmission of various infectious diseases of bovines, which emphasizes the need to develop and implement tailored extension programs in Pakistan to control contagious diseases of animals and to improve the profitability of small-scale dairy farmers.

## Introduction

The farming of livestock animals represents a crucial source of food to almost a billion people worldwide (1, 2). Milk is a major commodity, obtained from ~123 million dairy farms globally, maintained predominantly in mixed crop-livestock and pastoral systems in Asia and Africa, respectively (3–5). Estimates show that 68% of these farms are present in the Indian subcontinent, with >80% of them being small-scale operations (5, 6). Animal health issues are a major constraint to milk production, particularly for small-scale dairy farmers, due to poor disease diagnosis and monitoring and a limited understanding of disease epidemiology (7, 8). A similar situation exists in Pakistan, where the livestock sector plays a vital role in promoting socioeconomic development in rural areas. Approximately eight million families, with a total of 30–35 million people in rural populations, are involved in livestock production activities, which contribute to 35–40% of their annual income (9, 10).

Pakistan is the third-largest milk-producing country in the world and has 47.8 million cattle (*Bos indicus* and *Bos taurus*) and 40 million water buffaloes (*Bubalus bubalis*) (10, 11). Dairy animals are reared in four milk production systems: (1) rural subsistence smallholdings; (2) rural market-oriented smallholdings; (3) rural commercial dairy farms; and (4) peri-urban commercial dairy farms (12). Small-scale dairy farms (rural subsistence and rural market-oriented) with less than 10 animals per herd comprise > 90% of the total number of bovines in Pakistan and are predominantly in two provinces—Punjab and Sindh (13). The average milk yield per lactation on these small-holder farms is ~1,200 liters for buffaloes and 1,000 liters for cows, which is almost half of the national herd average for both species (14, 15).

The productivity of the Pakistani dairy sector is affected by constraints including nutrition, husbandry and health as well as limited access to vaccines and veterinary extension services (16, 17). The major bovine diseases/syndromes reported from Pakistan include mastitis, foot-and-mouth disease (FMD), hemorrhagic septicemia, blackleg (or black quarter) caused by *Clostridium chauvoei*, internal and external parasitic diseases and hemoglobinuria (14, 16, 18–21). In Pakistan, the annual losses due to animal diseases are reported to be US$ 200 million (8). By comparison, in India, Birthal et al. (22) recorded an annual loss of US$ 23.6 million (1.8 billion Indian rupees) to farmers, ascribed to various animal diseases and an annual loss of US$ 430 million due to FMD alone (8, 23). These losses could be reduced by adapting proper prevention and control measures against important diseases in developing countries. However, due to poor surveillance systems, there is little information on the incidence, distribution and dynamics of animal diseases in South Asian countries (8). The productivity of dairy animals on small-scale farms can be sub-optimal due to poor husbandry practices and limited resources, such that these farms can pose a greater biosecurity risk for the spread of livestock and zoonotic diseases compared with commercial dairy farms (24). Thus, it is important to investigate the knowledge base and practices of small-scale farmers regarding animal-health issues to prevent losses as well as to identify and mitigate biosecurity risks.

Undertaking studies in resource-poor communities is usually challenging because of their remote locations, limited infrastructure and challenges associated with data and sample collection as well as sample transport and storage (25). Additionally, without a long-term relationship with target communities, a lack of confidence about governmental authorities can also be a challenge in such studies (25). Despite these challenges, epidemiological investigations of livestock diseases should actively involve farmers to assess their knowledge and understanding of key issues and evaluate needs in local communities (26, 27). Interviews, surveys and participatory epidemiological (PE) tools assist such studies.

The PE approach includes participatory rural appraisal (such as informal interviews, visualization and mapping, scoring and ranking) combined with conventional epidemiological methods (28). PE tools should reduce non-sampling errors recognized in questionnaire surveys, such as poor responses to questions and interviewer errors (29), and they are inexpensive and convenient to conduct with illiterate participants, and provide validated results through triangulation of multiple methods (30, 31). This approach is now being widely used in parts of Africa and Asia for disease surveillance, prioritization, surveys, and control (32).

We elected to use a PE approach, to assess small-scale farmers' knowledge of bovine health and production (BH&P) issues compared with that of AHPs in five agroecological zones (AEZs) of Pakistan. The findings and conclusions from this study would address knowledge gaps and underpin future extension programs and biosecurity awareness campaigns for farmers, focused on increased farm productivity (33) as well as improved cooperation between farmers and AHPs.

## Materials and Methods

### Study Area

**Figure 1:**
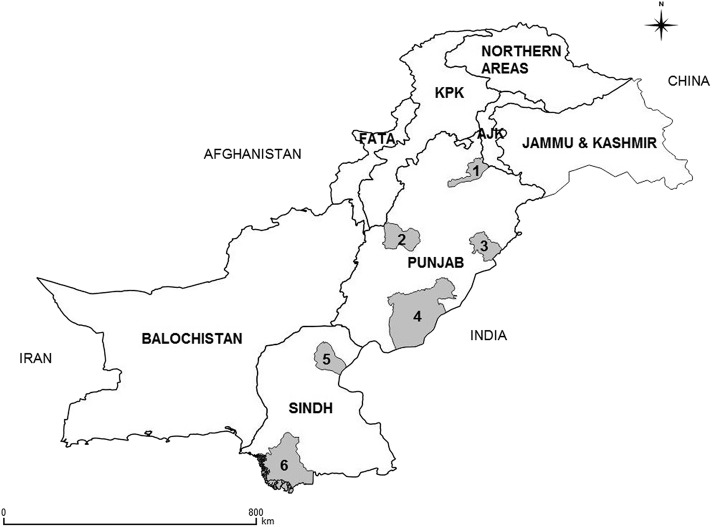
Map of Pakistan showing the locations of districts (gray-colored areas) included in the study. The names of the districts are Jhelum (1), Layyah (2), Okara (3), Bahawalpur (4), Sukkur (5), and Thatta (6). The map was created using QGIS v. 2.18.15.

Based on the physiography, land use, soil type, and climate, Pakistan is divided into 10 AEZs (34), and the distribution of bovines varies markedly across these zones (35). Small-scale dairy farms are mostly located in five of the 10 AEZs: Indus Delta, Northern Irrigated Plains, Arid (rain-fed), Sandy Desert, and Southern Irrigated Plains (35, 36). This study was conducted in four districts (Bahawalpur, Jhelum, Layyah, and Okara) of Punjab and the two districts (Sukkur and Thatta) of Sindh (Figure 1). These districts were selected based on operational convenience. However, each represented a different AEZ, except for the Arid zone, where two districts were selected (Jhelum and Layyah) to cover the diverse topography. The districts included are dominated by small-scale farmers, most of which are either landless or use rented land for agriculture, and mixed crop-livestock farming is usual. Men and women are mainly involved in livestock-related activities, with a varying degree of involvement of children. Many farmers with small-scale operations are resource-poor and, therefore, cannot afford to hire farm workers. Socioeconomic status varies particularly between provinces with least developed communities in districts of Sindh province. Descriptive statistics of temperature and the population of humans, cattle and buffalo per study district are given in Table 1.

**Table 1:** Demographic properties of districts included in the study.

**Province**	**District**	**Agroecological zones**	**Temperature (°*C*)^(37)(***Min***−***Max***)^**		**Population**		
			**Summer**	**Winter**	**Human (number)^(38)^**	**Bovine (million)^2^**	
						**Cattle**	**Buffalo**
Punjab	Bahawalpur	Sandy desert	39-46	1-7	3,668,106	0.79	0.81
	Jhelum^1^	Arid	38-45	0-6	1,222,650	0.24	0.19
	Layyah^1^	Arid	38-45	0-6	1,824,230	0.84	0.41
	Okara	Northern irrigated plains	39-46	1-6	3,039,139	0.47	1.18
Sindh	Sukkur	Southern irrigated plains	30-50	0-12	1,487,903	0.31	0.26
	Thatta	Indus delta	34-45	5-20	979,817	0.59	0.49

1 *Two districts were selected from arid zone to cover geographic diversity of this zone*.

2 *Estimated population in 2016 based on inter-census growth rate 1996 & 2006*.

### Structure of Veterinary Services in Pakistan

The provincial governments in Pakistan are responsible for providing veterinary services. Each province has its livestock department with various directorates offering services, including breed improvement, animal health, research, extension and disease surveillance (39). Animal health professionals (AHP) include veterinarians and para-veterinary staff. There are veterinary hospitals, dispensaries and centers, which provide veterinary services to livestock farmers in most parts of the country. They are supported by a disease diagnostic laboratory in each district; however, most of such laboratories are under-resourced, in terms of well-trained qualified staff and facilities. The extension services are poor, and less than 40% farmers have access to any livestock extension program. A veterinarian heads each veterinary hospital and is supported by a veterinary assistant as well as a technician to undertake artificial insemination in bovines (39, 40).

### Study Design

From September to November 2017, a cross-sectional study was conducted employing different PE methods. Following consultation with the Livestock Departments and the Dairy-Beef project, five villages were selected from each district. Eight to 10 small-scale dairy farmers (men) attended a facilitated group meeting (FGM) in each village; 30 FGMs were held in six districts. No women were involved in the FGMs, because of cultural and/or logistical issues. For AHPs, one FGM was conducted per district with each AHP group; 12 FGMs were held with AHPs (i.e., six with veterinary officers and six with veterinary assistants). No incentive was provided to farmers or AHPs for participation in any FGM.

### Participatory Epidemiology Methods Used

Four standard PE methods (simple ranking, pairwise ranking, constraint impact matrix scoring and constraint profiling) (29, 41–44) were used to assess the knowledge of dairy farmers and AHPs on the major constraints to BH&P. Simple and pairwise rankings were used as triangulation to validate the list of top constraints in all FGMs. Constraint impact matrix scoring was used to assess the impact of identified constraints on key health and production indicators. Constraint profiling informed about participants' knowledge about epidemiological aspects of the constraints. All FGMs were conducted in local languages.

The core research team consisted of two veterinarians and one veterinary assistant. One of the veterinarians acted as a facilitator, while the other recorded information on a board displayed at an appropriate distance from the participants. The veterinary assistant helped to organize FGMs and liaise with farmers. The FGMs were not voice-recorded to avoid any biases, because participants are usually reluctant to speak when they are audio- and/or video-recorded, despite them being assured of privacy and confidentiality. The checklist of topics and PE techniques were pre-tested before the FGMs in a pilot study and the data were excluded from the final study.

For each method, cross-checking and probing were carried out to: (1) validate the information, (2) ensure that the participants understood different items to be scored or ranked, and (3) ensure that enough information was gathered on each topic. During each activity, open-ended questions were asked to cross-examine the responses. However, utmost care was taken not to lead the participants to a specific health or production constraint. In each FGM, the participants were given time to discuss. If there was confusion about an issue or irrelevant discussion begun, the facilitator intervened through open-ended questions to guide the discussion, without forcing a consensus. The ranking, scoring and other aspects were recorded on charts. The same set of questions was used in all FGMs. Following each PE activity, the interview team met and reviewed the notes of each discussion to ensure that they were recorded objectively.

### Simple Ranking

Following a brief introduction of the team members and the objective of the study, participants were asked to name the important BH&P constraints causing high losses. In cases where the participants mentioned syndromes/symptoms as a constraint, probing, open-ended questions were asked to identify the local name of the constraint, but care was taken not to guide them to a particular BH&P constraint name. When the constraint name was not identified, further information was collected about the constraint until a proper diagnosis was reached. The constraint names were rechecked with the local veterinarian or para-veterinary staff at the end of the FGM. Once the constraints list was obtained, participants were asked to rank each constraint according to its perceived importance (i.e., caused losses). This activity produced a list of constraints (mean: 9, range: 6–12) based on their perceived importance (ranked from 1 to 12, where 1 = most important and 12 = least important).

### Pairwise Ranking and Comparison of Constraints to Bovine Health and Production

Pairwise rankings were carried out by comparing each constraint (already identified during simple ranking) individually with all other constraints. The simple ranking list of BH&P constraints was read aloud, and participants were asked again if they wanted to make any changes to the ranking order. Once re-validated, the top five constraints were taken from the list for pairwise ranking. The recorder sketched a 5 × 5 grid on a chart. The facilitator named the first constraint and asked participants to compare it with each of the remaining constraints, with supporting questions: (1) “Which of these two constraints is more important?”; (2) “Why is it more important?”; and (3) “Describe any differences/similarities between the two constraints.” The recorder documented the constraint that was indicated as most important by participants. The total score (0-4) for each constraint in a group was recorded, with 0 being the least important and 4 the most important. This provided pairwise ranks for each constraint on a scale of 1 to 5, with 1 as the most important and 5 as the least important. The median scores for each constraint given by the 30 farmer groups and the 12 AHP groups were then used to establish an overall ranking.

### Constraint Matrix Scoring

For each group discussion with farmers as well as AHPs, the five top-ranked BH&P constraints based on the pairwise ranking were scored against clinical and production-related indicators. These indicators were obtained during the pairwise comparisons in the pilot activity and included the impact on milk, meat, cost of treatment, morbidity and mortality. Indicators were written along the *y*-axis of a two-dimensional grid and the constraints along the *x*-axis. The facilitator asked the participants to distribute and/or assign a total of 10 small sticky dots to the five indicators for each of the constraints. Indicators and constraints were stated in the local language and explained to the participants when required. Participants were given time to develop consensus for voting, and once all the participants agreed on the number of dots for each indicator, the sticky dots were pasted on the chart in corresponding boxes. The sticky dots were counted in each of the corresponding boxes for each indicator giving the impact score. The scoring procedure was repeated until all the indicators had been scored against each constraint.

### Constraint Profiling

This activity was conducted to assess the participants' knowledge about the identified BH&P constraints. The recorder wrote the first constraint on a chart, and the facilitator asked open-ended questions about the descriptors of a constraint, including local name(s), cause, clinical signs, risk factors, the season of occurrence, susceptible age and animal species involved, treatment and vaccine availability/schedule. Responses were recorded in the corresponding boxes, and the same steps were repeated for all the constraints in order of their pairwise ranks. Notes from the discussion were recorded separately.

### Data Analyses

Data from all FGMs were entered into a spreadsheet (Microsoft Excel 2016). Kendall's coefficient of concordance (*W*) was used to quantify the level of agreement for pairwise rankings of BH&P constraints among farmers and AHPs at the district as well as provincial levels. An agreement was assessed as weak, moderate and strong for values of *W* under 0.26, between 0.26 and 0.38 and greater than 0.38, respectively (45). To determine the overall association between constraint impact score (as the outcome variable) and the health and production indicators (as an explanatory variable), we developed a fixed-effects proportional odds (ordinal) logistic regression model. The proportional odds logistic regression models were estimated using the contributed R (46) packages ordinal (47), RVAideMemoire (48), emmeans (49), and lattice (50). See Supplementary File S1 (R code), Supplementary File S2 (Farmer FGM data), and Supplementary File S3 (AHP FGM data) used for the analyses.

### Ethics Approval

This study was approved by the Veterinary and Agricultural Sciences Human Ethics Advisory Group, The University of Melbourne (Ethics ID: 1748953). All activities involving human participants followed the ethical standards of protecting the rights and welfare of participants. Data were collected after the participants gave consent.

## Results

### Demographics of Participants

The total number of participants included in this study was 277 (range: 8–10) and 83 (range: 5–8) in farmer and AHP FGMs, respectively. The farmers were adult men and mostly without any formal education; however, no data were recorded on the age and education of farmers. The AHPs included adult men (*n* = 79) and women (*n* = 4).

### Simple Ranking

**Figure 2:**
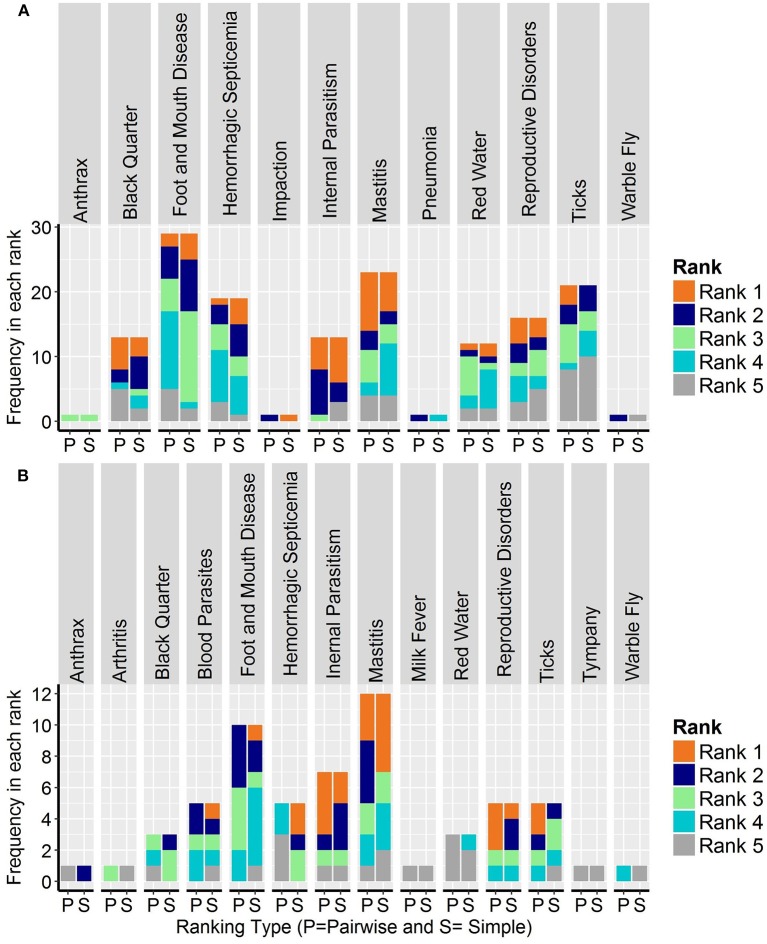
Grouped stacked bar plots of simple and pairwise rankings of major bovine health and production constraints by farmers **(A)** and animal health professionals **(B)**.

Simple ranking of BH&P constraints showed that FMD, mastitis, ticks, hemorrhagic septicemia, reproductive disorders, blackleg, and internal parasites were perceived to be important constraints by both farmers and AHPs (Figure 2). Farmers ranked FMD, mastitis, hemorrhagic septicemia, reproductive disorders and ticks as major constraints in all AEZs. Similarly, redwater was also important in all AEZs, except the southern irrigated zone. Overall, farmers ranked hemorrhagic septicemia as a major constraint due to higher mortality associated with this disease, particularly in southern irrigated plains and Indus delta zones (Figure 2A). FMD was ranked among the top-five constraints due to its relatively high and unpredictable incidence and recurrent outbreaks. However, it was mostly ranked 2nd or 3rd due to associated low mortality and self-recovery. Mastitis was considered as one of the leading causes of low milk yield, high treatment costs and poor prognosis as well as the reduced value of the animal. Tick infestation was listed as one of the important issues due to its negative impact on the growth and production of animals, particularly during the summer season. Similarly, farmers cited blackleg as the cause of high mortality in the arid, sandy desert and southern irrigated zones. Reproductive disorders including anestrus, prolapse and repeat breeding were also considered as economically important constraints by the farmers (Figure 2A).

AHPs ranked mastitis, FMD, internal parasites, hemorrhagic septicemia, blood parasites, reproductive disorders, ticks and blackleg among the top BH&P constraints (Figure 2B). They believed that mastitis was the most important health issue due to the low treatment success rate, leading to decreased milk production and reduced value of the animals. Despite mass vaccination campaigns against FMD by the provincial livestock departments, this disease was considered as the second major bovine health issue by AHPs. Internal parasites, mainly caused by liver fluke, were also of major concern due to their negative impact on an animal's body condition and milk yield. Hemorrhagic septicemia was considered a major health issue in Sindh province, and blackleg outbreaks were reported in arid and sandy desert zones of the Punjab province. AHPs also ranked ticks and tick-borne pathogens (i.e., *Anaplasma* spp., *Babesia* spp., and *Theileria* spp.) among major bovine health issues (Figure 2B). In contrast to farmers, AHPs mentioned that blood parasites were important in the northern irrigated, arid, and Indus delta zones.

### Pairwise Ranking

The pairwise ranking results showed that farmers ranked blackleg high when compared with other constraints due to higher mortality rates associated with it (Figure 2A). Mastitis was ranked higher due to low milk yield, the high cost of treatment and the poor prognosis. Similarly, AHPs considered the economic impact and prevalence rates of various constraints while comparing and ranking BH&P constraints. This activity resulted in changes in the ranking of all major issues; however, the overall ranking remained the same among both farmers and AHP (compare simple and pairwise rankings given in both Figures 2A,B). Kendall's coefficient of concordance showed strong agreement for pairwise ranks of BH&P constraints among farmers from all districts (*W* = 0.625, *P* = 0.000), as well as districts in Punjab (*W* = 0.718, *P* = 0.000) and in Sindh (*W* = 0.783, *P* = 0.045). The data from AHP FGMs from all districts also showed strong agreement (*W* = 0.915, *P* = 0.033).

### Constraint Impact Matrix Scoring

**Figure 3:**
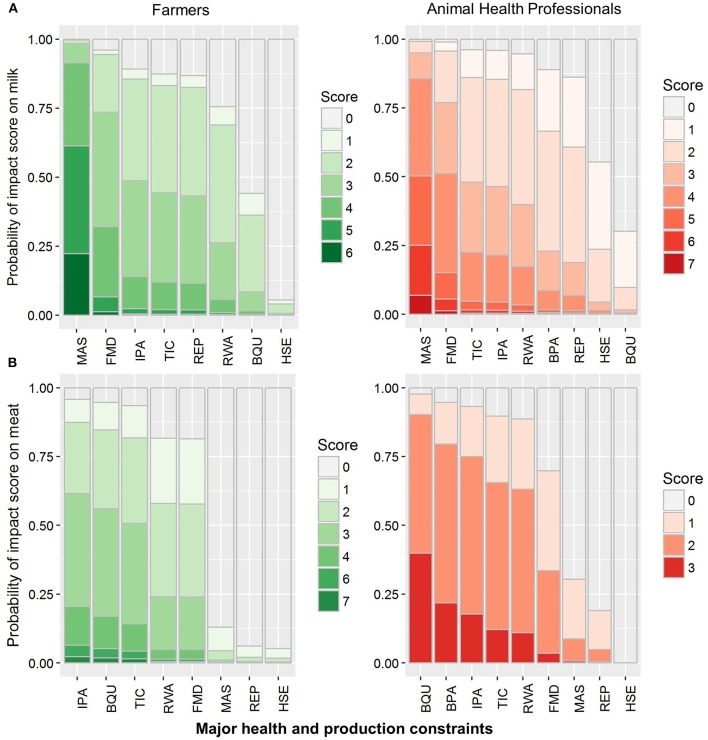
Grouped stacked bar plot of logistic regression of probability impact score of bovine health and production constraints on milk **(A)** and meat **(B)** production in cattle and buffaloes perceived by small-scale dairy farmers and animal health professionals in Pakistan. Major bovine health and production constraints include blood parasites (BPA), blackleg (BQU), foot-and-mouth disease (FMD), hemorrhagic septicemia (HSE), internal parasites (IPA), mastitis (MAS), Redwater (RWA), reproductive disorders (REP), and ticks (TIC).

This method was used to compare the impact of major BH&P constraints on each of the five identified indicators on the livelihood of small-scale dairy farmers. Ordinal logistic regression of impact scores from both farmers and AHPs indicated that milk production was perceived to be severely affected by mastitis and FMD (Figure 3A). Farmers perceived that mastitis affecting one udder quarter could lead to an almost 25% reduction in milk yield and would also decrease the value of an animal, and FMD outbreaks impacted at least one complete lactation in a herd. Farmers thought that blackleg was the main issue for the low quality and yield of meat, while internal parasites, ticks, and redwater caused weight loss and reduced growth rate (Figure 3B). Furthermore, farmers believed that ticks suck blood while internal parasites utilize animal's intestinal food, thereby leading to weight loss (Figure 3B).

**Figure 4:**
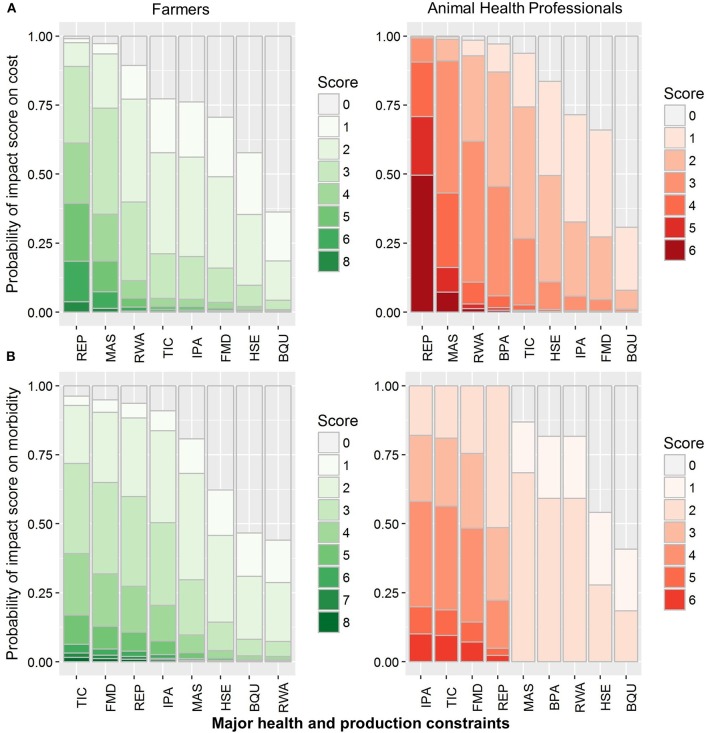
Grouped stacked bar plot of logistic regression of probability impact score on the cost of production **(A)** and morbidity caused by health and production constraints **(B)** in cattle and buffaloes perceived by small-scale dairy farmers and animal health professionals in Pakistan. Major bovine health and production constraints include blood parasites (BPA), blackleg (BQU), foot-and-mouth disease (FMD), hemorrhagic septicemia (HSE), internal parasites (IPA), mastitis (MAS), Redwater (RWA), reproductive disorders (REP), and ticks (TIC).

The cost was the third indicator for assessing the impact of BH&P constraints and the participants were asked about the economic impact (i.e., loss of income) and the expenses of treating a condition/constraint. Mastitis, redwater and reproductive disorders were considered as expensive constraints by both farmers and AHPs, whereas AHPs also included blood parasites in the list (Figure 4A). Both farmers and AHPs thought that mastitis required costly and prolonged therapy with little success, while reproductive disorders (anestrus in buffaloes and repeat breeding in cattle) caused losses to the small-scale dairy farmers in the form of increased calving interval, leading to higher costs involved in the feeding of dry animals. The fourth indicator was morbidity associated with a constraint/condition, and both groups identified that FMD, internal parasites, reproductive disorders (repeat breeding) and ticks had high morbidity (Figure 4B). According to farmers, FMD outbreaks occur biannually, and tick prevalence was usually higher during the summer season while internal parasites and reproductive disorder were prevalent throughout the year.

**Figure 5:**
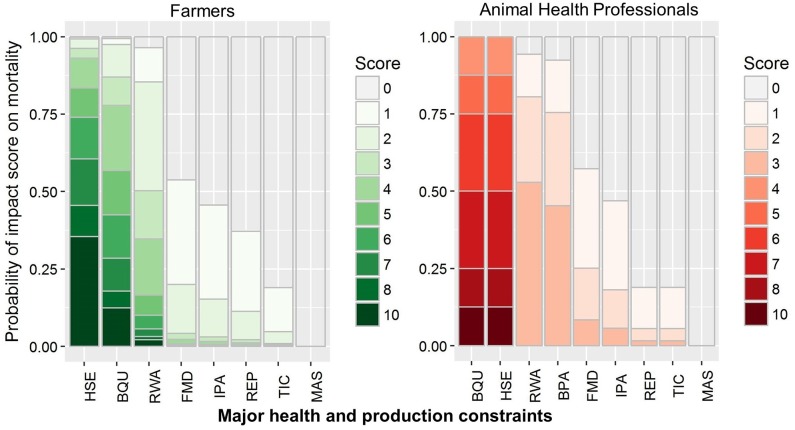
Grouped stacked bar plot of logistic regression of probability impact score on mortality caused by health and production constraints in cattle and buffaloes perceived by small-scale dairy farmers and animal health professionals in Pakistan. Major bovine health and production constraints include blood parasites (BPA), blackleg (BQU), foot-and-mouth disease (FMD), hemorrhagic septicemia (HSE), internal parasites (IPA), mastitis (MAS), Redwater (RWA), reproductive disorders (REP), and ticks (TIC).

Both farmers and AHPs commented that blackleg and hemorrhagic septicemia were responsible for most mortalities (Figure 5). Further probing showed that, although the prevalence was much lower for blackleg, it was still considered as a major life-threatening issue for animals, due to its high case fatality rate. Likewise, even though hemorrhagic septicemia had been controlled in most of the areas through the extensive use of vaccination and antibiotics, it was still a cause of high mortality. Farmers also mentioned redwater as a cause of high mortality, particularly in buffaloes. Further questions determined that both post-parturient hemoglobinuria as well as hemoglobinuria caused by babesiosis, were classified as redwater by farmers as well as AHPs.

### Constraint Profiling

This method was used to assess the participants' knowledge of important health and production issues, their seasonal occurrence and the relevant epidemiological aspects (Table 2). In Punjab province, farmers were able to describe key clinical signs of BH&P constraints while in Sindh province, farmers provided limited information on this aspect (such as fever in FMD, difficult breathing in hemorrhagic septicemia and reduced milk in mastitis). Overall, according to farmers, ethnoveterinary medicines were always the first line of treatment. In case of failure, they themselves used allopathic drugs, following advice provided by AHPs. Farmers perceived that their animals got infected with FMD after either being in direct contact or close to infected animals, although their knowledge about risk factors of FMD was limited.

**Table 2:** Description of local names and clinical signs of major bovine health and production constraints described by farmers and animal health professionals.

**Constraint**	**Local name(s)**	**Clinical signs**	**Season of occurrence**
Anestrus	Wohry Nathi	Anestrus and frequent vaginal discharge	Year round
Anthrax	Kaary Wa	Sudden death and oozing of blood from natural orifices	Year round
Blackleg	Chaurry Maar, Garri, Goli Maar, Karo Wah Tangwari, Tangwara	Discoloration of meat (black), crepitation sound from rear leg muscles, fever, a hot area around swollen areas, lameness and sudden death	Apr-Aug
Foot-and-mouth disease	Munh Khur, Samarro, Muharro, Bhagiyo	Anorexia, drooling of saliva, fever, high mortality in calves, lameness, loss of production and vesicles on feet and in mouth	Feb-Apr, Sep-Nov
Hemorrhagic septicemia	Gal Ghotu, Ghand, Ghogho, Ghanday	Coughing, difficulty in breathing, fever, swelling of the neck region and sudden death	Jul, Aug, Dec
Internal parasites	Keeray, aig (Liver Fluke)	Diarrhea, hair loss, weakness, lacrimal discharge, loss of production, pendulous abdomen and submandibular edema	Year round
Mastitis	Angiyari, Changiyari, Munh Sarri, Phikriyo, Saaru,	Blood and clots in milk, fever, teat blockage/fibrosis, loss of production, pus in milk, salty taste, swollen udder/teats and ulcers on teats	Year round
Redwater (hemoglobinuria)	Ratt Mutra, Sarkan, Saro	Blood in urine, constipation, frequent defecation (in small amounts), fever, loss of appetite, a low temperature of hindquarters	Jan, Feb, Aug, Sep
Repeat breeding	Phiraal	Repeated heat signs, increased vaginal discharge and a very high or low body condition score	Year round
Ticks	Chichar, Katway	Anemia, rough body skin, itching, skin lesions, ticks visible with naked eye and lassitude	Apr-Aug

Discussion on epidemiological aspects of mastitis revealed that infectious agents, unhygienic conditions and incorrect milking practices were the main contributing factors. For tick infestation, hot season, grazing, and lack of backyard poultry and water ponds were mentioned as the main risk factors. Tick control methods varied from region to region and mainly involved manual removal of ticks and burning, application of used engine oil, diesel, petrol and taramira oil (Eruca sativa) on animal's body besides periodical use of acaricides (such as avermectins, cypermethrin, trichlorofon). Hemorrhagic septicemia was identified as a disease with the highest mortality rate in young animals (6–24 months) during the rainy season. Hemorrhagic septicemia was mainly reported in both districts (Sukkur and Thatta) of Sindh due to the lack of vaccination. The main reproductive disorders were anestrus, repeat breeding and prolapse both in cattle and buffaloes. Redwater (hemoglobinuria) was ascribed to parturition, phosphorus deficiency and tick-borne pathogens.

Constraint profiling activity was also repeated with AHP to cross-examine farmer knowledge, and a strong agreement was found in responses between both groups. However, understandably, AHPs had better veterinary science knowledge about all aspects (causes, risk factors, clinical signs, treatment) of BH&P constraints compared with the farmers. Within AHPs, the knowledge level was comparable, but veterinary officers had a sound background in veterinary medicine as compared to veterinary assistants who possessed a comparatively better understanding of local names of constraints.

## Discussion

This study provides a detailed account of the important BH&P constraints that affect the livelihoods of small-scale dairy farmers in Pakistan. It also gives insights into the knowledge of farmers and AHPs about these constraints across different AEZs of the country. Previously, PE tools have been used mainly for animal health investigations in pastoral communities (51–56). Pastoral communities are common in African countries whereas small-scale mixed crop-livestock farming is common in South Asian countries like Pakistan and India which have two-thirds of global dairy farms and contributing a quarter of global milk production (5, 57).

The findings of this study are likely to reflect the situation in other provinces and neighboring countries because of similar farming and education systems. Inter-group bias was minimized by using the same research team to conduct all FGMs, despite all efforts, some information might have been lost. As only male farmers were involved in FGMs, some of the important information from women and/or children might have been lost. Furthermore, no data were collected about farmers' age, economic status, education, and their level of involvement in livestock-related activities; hence, no conclusions can be drawn about differences resulting from these factors. As all participants were volunteers from diverse socioeconomic backgrounds from different districts, the findings of this study should be interpreted with caution. Furthermore, the selection of villages was not random in this study due to limitations in accessing distant locations. It is envisaged that these challenges will be considered in the design of future studies.

Blackleg, FMD, hemorrhagic septicemia, internal parasites, mastitis, ticks and reproductive disorders were the main BH&P constraints for small-scale dairy farmers. Ghaffar et al. (58) and Khan (59) used PE tools to investigate issues and infectious diseases of bovines in Punjab and found that FMD, hemorrhagic septicemia, blackleg and mastitis were the major issues. Our results from the southern irrigated zone (Sukkur, canal-irrigated) also showed that FMD and hemorrhagic septicemia were the most important infectious diseases of bovines. However, earlier studies did not focus on small-scale dairy farmers, and none of them investigated farmers' understanding of the constraints for the productivity of their animals, rather, their focus was on the surveillance of infectious or transboundary diseases of livestock. To the best of our knowledge, this is the first study that utilized four complementary PE techniques to investigate the knowledge of farmers and AHPs about BH&P constraints faced by small-scale dairy farmers in Pakistan.

We found that farmers prioritized constraints based on the mortality rate associated with the constraint/condition, whereas AHPs ranked constraints based on their economic importance. This finding is consistent with previous reports by Ali et al. (19) and Hussain et al. (60) from Pakistan and Chatikobo et al. (61) from Zimbabwe. This difference of prioritizing constraints could be because small-scale dairy farmers use bovine animals as their primary source of income and any bovine health constraint which leads to mortality in the herd would have impacted their income, thereby making them remember such constraints. Another plausible cause could be due to the lack of record keeping in small-scale dairy farming systems. Furthermore, it also means that farmers pay less attention to those health and production constraints which are subclinical and do not lead to mortality of animals. These constraints (e.g., ticks, tick-borne pathogens, internal parasites) could be crucial for improving the productivity of their animals. Future extension programs targeted at farmers should focus on educating them about endemic BH&P constraints which cause production losses round the year.

In Punjab province, farmers' description of important clinical signs of BH&P constraints matched with that given by AHPs. However, in Sindh province, farmers were unable to describe constraints/conditions clearly. This might be due to the lack of veterinary services, poor implementation of extension programs targeted at farmers, poor adoption rates of an existing extension program and lower education level of farmers, also identified by Ali et al. (62). Given that most of the small-scale dairy farmers in Sindh belong to very low-income communities where the role of women in livestock rearing is usually higher, men might possess less information about constraints to livestock production (63). Hence, the difference in responses by farmers from Punjab and Sindh could be due to the different levels of involvement of men in livestock-related activities, but no data were collected to support this observation.

In Pakistan, the livestock extension programs targeted at livestock farmers are outdated and are mostly male-oriented (64). Previous studies have shown a higher involvement of women in day-to-day animal-related activities which varied from region to region and were indirectly governed by farmers' economic status (40, 63, 65). Recently, Wynn et al. (40) reported that the current extension services in Pakistan cover only 40% of small-scale farmers, a majority of which are men who have a minimal role in day-to-day livestock activities and thus lead to poor adoption of extension messages. This is supported by Warriach et al. (17) who implemented the whole-family approach (by involving men, women and children) for the extension program in Pakistan and found higher adoption rates leading to better animal health and on-farm benefits. Therefore, a whole-family approach for extension services might be a better approach to educate small-scale dairy farmers about health and production constraints for their animals.

In this study, the small-scale dairy farmers used ethnoveterinary medicines as the first choice of treatment for their animals because of high cost and perceived inefficacy of allopathic drugs, and in some cases unavailability of veterinary services in remote areas. A similar situation exists in many developing countries where resource-poor farmers are unable to afford the costly allopathic drugs and therefore, choose ethnoveterinary medicines that are cheap, easily available and have been used over generations (66). However, some infectious diseases (such as FMD and hemorrhagic septicemia) cause epidemics and require the use of broad-spectrum antibiotics and vaccines for the treatment and prevention (66). Therefore, it is essential to develop integrated approaches to control BH&P constraints faced by small-scale dairy farmers where the ethnoveterinary medicines practices are validated and conserved, and allopathic drugs and vaccines are also appropriately prescribed by AHPs.

Farmers informed that the introduction of new animal and lack of vaccines were responsible for FMD and hemorrhagic septicemia, respectively. Other causes identified by farmers were unhygienic conditions (mastitis), grazing practices (ticks), poor-quality water (liver fluke), nutritional deficiencies (reproductive disorders), and calving (redwater) or unknown agents. Farmers were able to describe the seasonal occurrence of BH&P constraints. They were dependent upon government-funded vaccination schemes in Punjab whereas, in Sindh, such schemes are not common and small-scale dairy farmers in remote areas do not have access to such schemes or veterinary services. These results show that small-scale dairy farmers had a good understanding of the common BH&P constraints. However, our results are contrary to those of Arif et al. (67) who found that most of the farmers did not know about brucellosis and its zoonotic potential. This difference could be due to limited knowledge of farmers about zoonoses and their importance *versus* important BH&P constraints which directly affect their livelihoods.

FMD is a highly contagious disease, and despite the use of local and imported vaccines, it is the most prevalent disease of bovines in Pakistan (68–70). However, farmers ranked FMD second to hemorrhagic septicemia or blackleg because of low mortality rate, particularly in adult animals. Similar findings were reported by Bellet et al. (71) who found that farmers ranked FMD second to hemorrhagic septicemia as it caused lower mortality in Cambodia. Interestingly, in the Indus delta in Sindh, FMD is considered as a fever-syndrome as well as a sign of good fortune for the animals, and farmers inoculate their animals with saliva or soil from hooves of infected animals. Although this practice leads to active immunization, it may also lead to clinical disease due to pathogenic strains of the FMD virus. Another practice to control FMD in Punjab was keeping or burning a camel's bone in the herd as farmers believed that FMD did not affect camels. Although the dromedary camels are not susceptible to FMD and do not transmit it to other animals (72), there is no scientific evidence to support this myth of keeping or burning camel bones in the herd to prevent FMD. Ferrari et al. (73) and Jemberu et al. (74) estimated the economic impact of FMD in Pakistan and Ethiopia, respectively, and found that high economic losses occurring mainly due to loss of milk yield could be prevented by adopting timely appropriate preventive measures such as vaccination and biosecurity measures.

Currently, Pakistan is at stage-2 of FMD Progressive Control Pathway (PCP) which commenced in 2008, in collaboration with FAO and OIE (75). The present study demonstrates that farmers usually lack knowledge about risk factors for transmission of FMD and engage in several risky practices. In order to advance to stage-3 of FMD PCP, an extensive farmer extension program should be implemented in the country, providing critical information on routes of transmission and risk factors. Additionally, designing and implementing an FMD simulation model would also be pivotal for controlling losses due to FMD in Pakistan and other endemic countries (76).

In this study, both farmers and AHPs ranked mastitis as one of the major causes of losses on small-scale dairy farms in Pakistan where it is highly prevalent in dairy animals (59, 77, 78). Small-scale dairy farmers treat mastitis with ethnoveterinary medicines such as garlic and red chili, and they do not contact the AHPs until the chronic stage of mastitis develops due to the high cost of allopathic drugs.

Gastrointestinal parasitism was also ranked as a major cause of economic losses to small-scale dairy farmers across all AEZs. Farmers reported liver fluke (*Fasciola hepatica* and *F. gigantica*) infestation with in both AEZs of Sindh province. This could be due to the location of these areas along the river-bank, which provide suitable environment for the intermediate host (snail) of liver flukes (79). In Sindh, small-scale dairy farmers have low income, and they cannot afford to deworm their animals for liver fluke. Like in Punjab, scheduled deworming programs for livestock in Sindh can help to reduce production losses associated with gastrointestinal parasitism in dairy animals.

Farmers and AHPs considered tick infestation as an important BH&P constraint in all AEZs. The clinical signs of tick infestation mentioned were visibility of ticks with the naked eye, general weakness of animals and skin lesions caused by bites. Farmers believed that tick infestation was endemic throughout the year due to inefficacy of available acaricides and production losses were caused mainly due to the blood sucked by ticks. Previously, poor husbandry practices have been found to be associated with tick infestation on small-scale dairy farms (80–82). Manual grooming is a widely practiced method by small-scale farmers for tick control; however, it is time-consuming and bears a risk of the transmission of zoonotic diseases such as Crimean-Congo hemorrhagic fever. Farmers did not perceive tick-borne pathogens as a threat (in contrast to AHPs) to their animals, although anaplasmosis, babesiosis and theileriosis are endemic bovine tick-borne diseases (TBD) in Pakistan (83). Recent investigations have demonstrated that bovine ticks carry a diverse array of pathogens, some of which can be zoonotic (84). Losses due to TBDs could be very high in Pakistan, particularly on resource-poor farms. Recently, Sungirai et al. (85) investigated farmers' perceptions about TBDs in Zimbabwe and found that 67% of farmers were able to describe TBDs with signs. The high level of TBDs awareness resulted in farmers engaging in precautionary measures such as regular acaricidal dipping of their animals. Similarly, small-scale dairy farmers in Pakistan should be provided with information about the role of ticks as vectors and the adverse impact of ticks and TBDs on the health of their animals.

## Conclusion

Despite the pivotal contribution of bovines to the food and livelihood of small-scale dairy farmers of Pakistan, the productivity of cattle and buffaloes is limited by health and production issues, including blackleg, FMD, hemorrhagic septicemia, mastitis, ecto- and endo-parasites, and reproductive disorders. Farmers possess good knowledge about these constraints; however, they lack sufficient information about the risk factors about important BH&P constraints. There is no variation of constraints between/among different AEZs within the same province. In most cases, traditional (herbal) remedies are used to treat BH&P constraints. Farmers prioritize constraints that cause high mortalities, whereas AHPs consider economic losses for ranking constraints. This study emphasizes the need for simple extension programs using a whole-family approach by covering all critical aspects of BH&P constraints to prevent economic losses to small-scale dairy farmers.

## Data Availability Statement

All datasets generated for this study are included in the article/Supplementary Material.

## Ethics Statement

The studies involving human participants were reviewed and approved by Veterinary and Agricultural Sciences Human Ethics Advisory Group, The University of Melbourne (Ethics ID: 1748953). Written informed consent for participation was not required for this study in accordance with the national legislation and the institutional requirements.

## Author Contributions

AJ, AG, RG, DM, and MS designed the study. AG, MB, and AK conducted the study. HW provided logistic support for the field work. AG, MS, DM, and AJ analyzed the results. AG and AJ drafted the manuscript. All authors contributed to the manuscript revision, read and approved the manuscript.

## Conflict of Interest

The authors declare that the research was conducted in the absence of any commercial or financial relationships that could be construed as a potential conflict of interest.

## Abbreviations

AEZ: agroecological zone
AHP: animal health professionals
BH&P: bovine health and production
FMD: foot-and-mouth disease
PE: participatory epidemiology.
