# Supplementary material for: A Participatory Investigation of Bovine Health and Production Issues in Pakistan
Source: Front Vet Sci. 2020 May 6;7:248. doi: 10.3389/fvets.2020.00248 (PMC7218055; doi:10.3389/fvets.2020.00248)
Supplement: Supplementary file 3 [file Data_Sheet_3.docx]

*Supplementary Material*

R Code for Regression Model

##Download required libraries##

library(ordinal) # for ordinal logistic models

library(RVAideMemoire) # easy way to get model predictions for ordinal model

library(emmeans) # also needed for model predictions

library(lattice) # for graphic

library(ggplot2)

library(RColorBrewer)

##prepare data files##

fpra <- read.csv("Supplementary_File_S2.csv")

table(fpra$dis)

disfreq <- table(fpra$dis)

dis.sel <- rownames(disfreq)[disfreq > 1]

fpra <- fpra[fpra$dis %in% dis.sel,]

fpra$dis <- factor(fpra$dis)

table(fpra$dis)

fpra$adis[fpra$dis == "Black quarter"] <- "BQU"

fpra$adis[fpra$dis == "FMD"] <- "FMD"

fpra$adis[fpra$dis == "Haemorrhagic septicaemia"] <- "HSE"

fpra$adis[fpra$dis == "Internal parasitism"] <- "IPA"

fpra$adis[fpra$dis == "Mastitis"] <- "MAS"

fpra$adis[fpra$dis == "Red water"] <- "RWA"

fpra$adis[fpra$dis == "Reproductive disorders"] <- "REP"

fpra$adis[fpra$dis == "Ticks"] <- "TIC"

fpra$milk <- factor(fpra$milk)

fpra$meat <- factor(fpra$meat)

fpra$cost <- factor(fpra$cost)

fpra$morbidity <- factor(fpra$morb)

fpra$mortality <- factor(fpra$mort)

# ---------------------------------------------------------------------------------------------------------------

ahpr <- read.csv("Supplementary_File_S3.csv")

table(ahpr$dis)

disfreq <- table(ahpr$dis)

dis.sel <- rownames(disfreq)[disfreq > 1]

ahpr <- ahpr[ahpr$dis %in% dis.sel,]

ahpr$dis <- factor(ahpr$dis)

table(ahpr$dis)

ahpr$adis[ahpr$dis == "Black quarter"] <- "BQU"

ahpr$adis[ahpr$dis == "Blood parasites"] <- "BPA"

ahpr$adis[ahpr$dis == "FMD"] <- "FMD"

ahpr$adis[ahpr$dis == "Haemorrhagic septicaemia"] <- "HSE"

ahpr$adis[ahpr$dis == "Internal parasitism"] <- "IPA"

ahpr$adis[ahpr$dis == "Mastitis"] <- "MAS"

ahpr$adis[ahpr$dis == "Red water"] <- "RWA"

ahpr$adis[ahpr$dis == "Reproductive disorders"] <- "REP"

ahpr$adis[ahpr$dis == "Ticks"] <- "TIC"

ahpr$milk <- factor(ahpr$milk)

ahpr$meat <- factor(ahpr$meat)

ahpr$cost <- factor(ahpr$cost)

ahpr$morbidity <- factor(ahpr$morb)

ahpr$mortality <- factor(ahpr$mort)

#====================================================================

# Milk probability calculation

fpr.clmilk <- clm(milk ~ adis, data = fpra)

# Model-based probabilities:

emilk <- emmeans(fpr.clmilk, ~ adis | cut, mode = "linear.predictor")

predmilk <- rating.emmeans(emilk, type = "prob")

head(predmilk)

# We want the order of diseases (listed on the horizontal axis of the stacked bar chart) to be sorted in order of Rating = 0:

sort <- predmilk[predmilk$Rating == 0,]

sort <- sort[sort.list(sort$Prob, decreasing = FALSE),]

sort$xpos <- 1:nrow(sort)

sort

# Update data frame predmilk with xpos:

predmilk$xpos <- sort$xpos[match(predmilk$adis, sort$adis)]

predmilk$Rating <- factor(predmilk$Rating, levels = 0:6)

predmilk$xpos <- factor(predmilk$xpos, levels = sort$xpos, labels = sort$adis)

mypalette <- rev(c(rev(brewer.pal(6, "Greens")), "transparent"))

# Stacked bar chart using ggplot2:

fpr.milk <- ggplot(data = predmilk, aes(x = xpos, y = Prob, fill = Rating)) +

geom_bar(stat = "identity", col = "grey") +

xlab("Health issue") +

ylab("Probability of impact on milk") +

scale_fill_manual(values = mypalette) +

theme(axis.text.x = element_text(size = 10, angle = 90), axis.title.x = element_text(size = 12)) +

theme(axis.text.y = element_text(size = 10, angle = 0), axis.title.y = element_text(size = 12)) +

theme(legend.text = element_text(size = 10), legend.title = element_text(size = 12)) +

labs(fill = "Score")

windows(); fpr.milk

#------------------------------------------------------------------------------------------------------------------

ahp.clm <- clm(milk ~ adis, data = ahpr)

# Model-based probabilities:

emilk <- emmeans(ahp.clm, ~ adis | cut, mode = "linear.predictor")

predmilk <- rating.emmeans(emilk, type = "prob")

head(predmilk)

# We want the order of diseases (listed on the horizontal axis of the stacked bar chart) to be sorted in order of Rating = 0:

sort <- predmilk[predmilk$Rating == 0,]

sort <- sort[sort.list(sort$Prob, decreasing = FALSE),]

sort$xpos <- 1:nrow(sort)

sort

# Update data frame predmilk with xpos:

predmilk$xpos <- sort$xpos[match(predmilk$adis, sort$adis)]

predmilk$xpos <- factor(predmilk$xpos, levels = sort$xpos, labels = sort$adis)

predmilk$Rating <- factor(predmilk$Rating, levels = 0:7)

mypalette <- rev(c(rev(brewer.pal(7, "Reds")), "transparent"))

# Stacked bar chart using ggplot2:

ahp.milk <- ggplot(data = predmilk, aes(x = xpos, y = Prob, fill = Rating)) +

geom_bar(stat = "identity", col = "grey") +

xlab("Health issue") +

ylab("Probability of impact on milk") +

scale_fill_manual(values = mypalette) +

theme(axis.text.x = element_text(size = 10, angle = 90), axis.title.x = element_text(size = 12)) +

theme(axis.text.y = element_text(size = 10, angle = 0), axis.title.y = element_text(size = 12)) +

theme(legend.text = element_text(size = 10), legend.title = element_text(size = 12)) +

labs(fill = "Score")

windows(); ahp.milk

#------------------------------------------------------------------------------------------------------------------

# Meat probability calculation

fpr.clmeat <- clm(meat ~ adis, data = fpra)

# Model-based probabilities:

emeat <- emmeans(fpr.clmeat, ~ adis | cut, mode = "linear.predictor")

predmeat <- rating.emmeans(emeat, type = "prob")

head(predmeat)

# We want the order of diseases (listed on the horizontal axis of the stacked bar chart) to be sorted in order of Rating = 0:

sort <- predmeat[predmeat$Rating == 0,]

sort <- sort[sort.list(sort$Prob, decreasing = FALSE),]

sort$xpos <- 1:nrow(sort)

sort

# Update data frame predmeat with xpos:

predmeat$xpos <- sort$xpos[match(predmeat$adis, sort$adis)]

predmeat$Rating <- factor(predmeat$Rating, levels = 0:7)

predmeat$xpos <- factor(predmeat$xpos, levels = sort$xpos, labels = sort$adis)

mypalette <- rev(c(rev(brewer.pal(7, "Greens")), "transparent"))

# Stacked bar chart using ggplot2:

fpr.meat <- ggplot(data = predmeat, aes(x = xpos, y = Prob, fill = Rating)) +

geom_bar(stat = "identity", col = "grey") +

xlab("Health issue") +

ylab("Probability of impact on meat") +

scale_fill_manual(values = mypalette) +

theme(axis.text.x = element_text(size = 10, angle = 90), axis.title.x = element_text(size = 12)) +

theme(axis.text.y = element_text(size = 10, angle = 0), axis.title.y = element_text(size = 12)) +

theme(legend.text = element_text(size = 10), legend.title = element_text(size = 12)) +

labs(fill = "Score")

windows(); fpr.meat

#------------------------------------------------------------------------------------------------------------------

ahp.clmeat <- clm(meat ~ adis, data = ahpr)

# Model-based probabilities:

ahpmeat <- emmeans(ahp.clmeat, ~ adis | cut, mode = "linear.predictor")

predmeat <- rating.emmeans(ahpmeat, type = "prob")

head(predmeat)

# We want the order of diseases (listed on the horizontal axis of the stacked bar chart) to be sorted in order of Rating = 0:

sort <- predmeat[predmeat$Rating == 0,]

sort <- sort[sort.list(sort$Prob, decreasing = FALSE),]

sort$xpos <- 1:nrow(sort)

sort

# Update data frame predmeat with xpos:

predmeat$xpos <- sort$xpos[match(predmeat$adis, sort$adis)]

predmeat$xpos <- factor(predmeat$xpos, levels = sort$xpos, labels = sort$adis)

predmeat$Rating <- factor(predmeat$Rating, levels = 0:3)

mypalette <- rev(c(rev(brewer.pal(3, "Reds")), "transparent"))

# Stacked bar chart using ggplot2:

ahp.meat <- ggplot(data = predmeat, aes(x = xpos, y = Prob, fill = Rating)) +

geom_bar(stat = "identity", col = "grey") +

xlab("Health issue") +

ylab("Probability of impact on meat") +

scale_fill_manual(values = mypalette) +

theme(axis.text.x = element_text(size = 10, angle = 90), axis.title.x = element_text(size = 12)) +

theme(axis.text.y = element_text(size = 10, angle = 0), axis.title.y = element_text(size = 12)) +

theme(legend.text = element_text(size = 10), legend.title = element_text(size = 12)) +

labs(fill = "Score")

windows(); ahp.meat

#------------------------------------------------------------------------------------------------------------------

library(gridExtra)

ahp.milkmeat <- windows(); grid.arrange(fpr.milk, ahp.milk, fpr.meat, ahp.meat, ncol = 2, nrow = 2)

#------------------------------------------------------------------------------------------------------------------

# Cost probability calculation

fpr.cost <- clm(cost ~ adis, data = fpra)

# Model-based probabilities:

ecost <- emmeans(fpr.cost, ~ adis | cut, mode = "linear.predictor")

predcost <- rating.emmeans(ecost, type = "prob")

head(predcost)

# We want the order of diseases (listed on the horizontal axis of the stacked bar chart) to be sorted in order of Rating = 0:

sort <- predcost[predcost$Rating == 0,]

sort <- sort[sort.list(sort$Prob, decreasing = FALSE),]

sort$xpos <- 1:nrow(sort)

sort

# Update data frame predcost with xpos:

predcost$xpos <- sort$xpos[match(predcost$adis, sort$adis)]

predcost$Rating <- factor(predcost$Rating, levels = 0:8)

predcost$xpos <- factor(predcost$xpos, levels = sort$xpos, labels = sort$adis)

mypalette <- rev(c(rev(brewer.pal(8, "Greens")), "transparent"))

# Stacked bar chart using ggplot2:

fpr.cost <- ggplot(data = predcost, aes(x = xpos, y = Prob, fill = Rating)) +

geom_bar(stat = "identity", col = "grey") +

xlab("Health issue") +

ylab("Probability of impact on cost") +

scale_fill_manual(values = mypalette) +

theme(axis.text.x = element_text(size = 10, angle = 90), axis.title.x = element_text(size = 12)) +

theme(axis.text.y = element_text(size = 10, angle = 0), axis.title.y = element_text(size = 12)) +

theme(legend.text = element_text(size = 10), legend.title = element_text(size = 12)) +

labs(fill = "Score")

windows(); fpr.cost

#------------------------------------------------------------------------------------------------------------------

ahp.cost <- clm(cost ~ adis, data = ahpr)

# Model-based probabilities:

emcost <- emmeans(ahp.cost, ~ adis | cut, mode = "linear.predictor")

predcost <- rating.emmeans(emcost, type = "prob")

head(predcost)

# We want the order of diseases (listed on the horizontal axis of the stacked bar chart) to be sorted in order of Rating = 0:

sort <- predcost[predcost$Rating == 0,]

sort <- sort[sort.list(sort$Prob, decreasing = FALSE),]

sort$xpos <- 1:nrow(sort)

sort

# Update data frame predcost with xpos:

predcost$xpos <- sort$xpos[match(predcost$adis, sort$adis)]

predcost$xpos <- factor(predcost$xpos, levels = sort$xpos, labels = sort$adis)

predcost$Rating <- factor(predcost$Rating, levels = 0:6)

mypalette <- rev(c(rev(brewer.pal(6, "Reds")), "transparent"))

# Stacked bar chart using ggplot2:

ahp.cost <- ggplot(data = predcost, aes(x = xpos, y = Prob, fill = Rating)) +

geom_bar(stat = "identity", col = "grey") +

xlab("Health issue") +

ylab("Probability of impact on cost") +

scale_fill_manual(values = mypalette) +

theme(axis.text.x = element_text(size = 10, angle = 90), axis.title.x = element_text(size = 12)) +

theme(axis.text.y = element_text(size = 10, angle = 0), axis.title.y = element_text(size = 12)) +

theme(legend.text = element_text(size = 10), legend.title = element_text(size = 12)) +

labs(fill = "Score")

windows(); ahp.cost

#-----------------------------------------------------------------------------------------------------------------

# Morbidity probability calculation

fpr.morbidity <- clm(morbidity ~ adis, data = fpra)

# Model-based probabilities:

emorbidity <- emmeans(fpr.morbidity, ~ adis | cut, mode = "linear.predictor")

predmorbidity <- rating.emmeans(emorbidity, type = "prob")

head(predmorbidity)

# We want the order of diseases (listed on the horizontal axis of the stacked bar chart) to be sorted in order of Rating = 0:

sort <- predmorbidity[predmorbidity$Rating == 0,]

sort <- sort[sort.list(sort$Prob, decreasing = FALSE),]

sort$xpos <- 1:nrow(sort)

sort

# Update data frame predmorbidity with xpos:

predmorbidity$xpos <- sort$xpos[match(predmorbidity$adis, sort$adis)]

predmorbidity$Rating <- factor(predmorbidity$Rating, levels = 0:8)

predmorbidity$xpos <- factor(predmorbidity$xpos, levels = sort$xpos, labels = sort$adis)

mypalette <- rev(c(rev(brewer.pal(8, "Greens")), "transparent"))

# Stacked bar chart using ggplot2:

fpr.morbidity <- ggplot(data = predmorbidity, aes(x = xpos, y = Prob, fill = Rating)) +

geom_bar(stat = "identity", col = "grey") +

xlab("Health issue") +

ylab("Probability of impact on morbidity") +

scale_fill_manual(values = mypalette) +

theme(axis.text.x = element_text(size = 10, angle = 90), axis.title.x = element_text(size = 12)) +

theme(axis.text.y = element_text(size = 10, angle = 0), axis.title.y = element_text(size = 12)) +

theme(legend.text = element_text(size = 10), legend.title = element_text(size = 12)) +

labs(fill = "Score")

windows(); fpr.morbidity

#------------------------------------------------------------------------------------------------------------------

ahp.morbidity <- clm(morbidity ~ adis, data = ahpr)

# Model-based probabilities:

emorbidity <- emmeans(ahp.morbidity, ~ adis | cut, mode = "linear.predictor")

predmorbidity <- rating.emmeans(emorbidity, type = "prob")

head(predmorbidity)

# We want the order of diseases (listed on the horizontal axis of the stacked bar chart) to be sorted in order of Rating = 0:

sort <- predmorbidity[predmorbidity$Rating == 0,]

sort <- sort[sort.list(sort$Prob, decreasing = FALSE),]

sort$xpos <- 1:nrow(sort)

sort

# Update data frame predmilk with xpos:

predmorbidity$xpos <- sort$xpos[match(predmorbidity$adis, sort$adis)]

predmorbidity$xpos <- factor(predmorbidity$xpos, levels = sort$xpos, labels = sort$adis)

predmorbidity$Rating <- factor(predmorbidity$Rating, levels = 0:6)

mypalette <- rev(c(rev(brewer.pal(6, "Reds")), "transparent"))

# Stacked bar chart using ggplot2:

ahp.morbidity <- ggplot(data = predmorbidity, aes(x = xpos, y = Prob, fill = Rating)) +

geom_bar(stat = "identity", col = "grey") +

xlab("Health issue") +

ylab("Probability of impact on morbidity") +

scale_fill_manual(values = mypalette) +

theme(axis.text.x = element_text(size = 10, angle = 90), axis.title.x = element_text(size = 12)) +

theme(axis.text.y = element_text(size = 10, angle = 0), axis.title.y = element_text(size = 12)) +

theme(legend.text = element_text(size = 10), legend.title = element_text(size = 12)) +

labs(fill = "Score")

windows(); ahp.morbidity

#------------------------------------------------------------------------------------------------------------------

library(gridExtra)

ahp.costmorb <- windows(); grid.arrange(fpr.cost, ahp.cost, fpr.morbidity, ahp.morbidity, ncol = 2, nrow = 2)

#------------------------------------------------------------------------------------------------------------------

# Mortality probability calculation

fpr.mortality <- clm(mortality ~ adis, data = fpra)

# Model-based probabilities:

emortality <- emmeans(fpr.mortality, ~ adis | cut, mode = "linear.predictor")

predmortality <- rating.emmeans(emortality, type = "prob")

head(predmortality)

# We want the order of diseases (listed on the horizontal axis of the stacked bar chart) to be sorted in order of Rating = 0:

sort <- predmortality[predmortality$Rating == 0,]

sort <- sort[sort.list(sort$Prob, decreasing = FALSE),]

sort$xpos <- 1:nrow(sort)

sort

# Update data frame predmortality with xpos:

predmortality$xpos <- sort$xpos[match(predmortality$adis, sort$adis)]

predmortality$Rating <- factor(predmortality$Rating, levels = 0:10)

predmortality$xpos <- factor(predmortality$xpos, levels = sort$xpos, labels = sort$adis)

mypalette <- rev(c(rev(brewer.pal(10, "Greens")), "transparent"))

# Stacked bar chart using ggplot2:

fpr.mortality <- ggplot(data = predmortality, aes(x = xpos, y = Prob, fill = Rating)) +

geom_bar(stat = "identity", col = "grey") +

xlab("Health issue") +

ylab("Probability of impact on mortality") +

scale_fill_manual(values = mypalette) +

theme(axis.text.x = element_text(size = 10, angle = 90), axis.title.x = element_text(size = 12)) +

theme(axis.text.y = element_text(size = 10, angle = 0), axis.title.y = element_text(size = 12)) +

theme(legend.text = element_text(size = 10), legend.title = element_text(size = 12)) +

labs(fill = "Score")

windows(); fpr.mortality

#------------------------------------------------------------------------------------------------------------------

ahp.mortality <- clm(mortality ~ adis, data = ahpr)

# Model-based probabilities:

emortality <- emmeans(ahp.mortality, ~ adis | cut, mode = "linear.predictor")

predmortality <- rating.emmeans(emortality, type = "prob")

head(predmortality)

# We want the order of diseases (listed on the horizontal axis of the stacked bar chart) to be sorted in order of Rating = 0:

sort <- predmortality[predmortality$Rating == 0,]

sort <- sort[sort.list(sort$Prob, decreasing = FALSE),]

sort$xpos <- 1:nrow(sort)

sort

# Update data frame predmortality with xpos:

predmortality$xpos <- sort$xpos[match(predmortality$adis, sort$adis)]

predmortality$xpos <- factor(predmortality$xpos, levels = sort$xpos, labels = sort$adis)

predmortality$Rating <- factor(predmortality$Rating, levels = 0:10)

mypalette <- rev(c(rev(brewer.pal(10, "Reds")), "transparent"))

# Stacked bar chart using ggplot2:

ahp.mortality <- ggplot(data = predmortality, aes(x = xpos, y = Prob, fill = Rating)) +

geom_bar(stat = "identity", col = "grey") +

xlab("Health issue") +

ylab("Probability of impact on mortality") +

scale_fill_manual(values = mypalette) +

theme(axis.text.x = element_text(size = 10, angle = 90), axis.title.x = element_text(size = 12)) +

theme(axis.text.y = element_text(size = 10, angle = 0), axis.title.y = element_text(size = 12)) +

theme(legend.text = element_text(size = 10), legend.title = element_text(size = 12)) +

labs(fill = "Score")

windows(); ahp.mortality

#====================================================================
